# Delayed Silicotuberculosis Diagnosis in a Young Worker: The Importance of Dust Control and Occupational Surveillance

**DOI:** 10.1155/crpu/2249648

**Published:** 2026-10-26

**Authors:** Richar Tomy Thendeyas, Daniel Maranatha, Nurul Atika

**Affiliations:** ^1^ Pulmonology and Respiratory Medicine Department Faculty of Medicine Airlangga University Dr. Soetomo General Academic Hospital Surabaya East Java Indonesia unair.ac.id

## Abstract

Silicosis is an irreversible occupational lung disease caused by inhaled crystalline silica and is a known risk factor for tuberculosis. We report a 26‐year‐old male construction worker with more than 10 years of unprotected silica dust exposure who presented with worsening dyspnea, chronic productive cough, and weakness. He had been diagnosed with pulmonary tuberculosis by GeneXpert and was receiving continuation‐phase antituberculosis treatment. Chest imaging showed bilateral fibroinfiltrative changes, fibroconsolidation, bronchial dilatation, pleural thickening, and calcified nodules in the right upper lobe, suggesting silicotuberculosis. Bronchoscopy was deferred due to high anesthetic risk, and open lung biopsy with wedge resection was performed. Histopathology showed chronic nonspecific inflammation, dust‐laden macrophages, multinucleated giant cells, and fibrosis, without alveolar proteinosis or classic silicotic nodules. Given his more than 10 years of unprotected occupational exposure to silica‐containing dust, confirmed pulmonary tuberculosis, and overlapping radiological and pathological abnormalities, the overall presentation was interpreted as silicotuberculosis with an accelerated clinical course. The patient received oxygen therapy, continued antituberculosis treatment, antibiotics, bronchodilator nebulization, mucolytic therapy, and chest physiotherapy. His symptoms improved, but he remained oxygen‐dependent during mobilization at discharge. He was subsequently readmitted in May 2024 with worsening respiratory symptoms and died during hospitalization. This case emphasizes the importance of occupational history and further evaluation for silicosis in young tuberculosis patients with persistent respiratory abnormalities and silica exposure.

## 1. Introduction

Silicosis is an occupational fibrotic lung disease caused by inhalation of respirable crystalline silica and remains one of the most important forms of pneumoconiosis worldwide. Despite longstanding prevention efforts, it continues to pose a substantial burden in low‐income and middle‐income settings, particularly among workers with prolonged exposure to silica‐containing dust. Disease progression may persist even after cessation of exposure, ultimately resulting in chronic inflammation, irreversible fibrosis, and long‐term functional impairment [1–4].

Silica exposure and silicosis are also well‐recognized risk factors for tuberculosis (TB). As a result, individuals with silicosis have a markedly greater risk of developing active TB than the general population, making silicotuberculosis an important clinical and public health concern, especially in TB‐endemic regions [2, 3, 5, 6].

The diagnosis of silicosis is usually established from a combination of relevant occupational exposure history, supportive radiologic findings, and exclusion of other causes of diffuse parenchymal lung disease. In practice, however, diagnostic evaluation can be challenging when the clinical and imaging features overlap with pulmonary TB, bacterial pneumonia, or other chronic lung disorders. In selected complex cases, histopathological or tissue‐based assessment may be necessary, particularly when less invasive diagnostic procedures are not feasible or fail to provide sufficient diagnostic certainty [2, 3, 7, 8].

We report the case of a 26‐year‐old male construction worker with more than 10 years of unprotected occupational exposure to silica‐containing dust and pulmonary TB, whose overall presentation was interpreted as silicotuberculosis with an accelerated clinical course. This case emphasizes the importance of thorough occupational history taking and recognition of the interacting contributions of silica exposure and TB in young workers with severe, rapidly progressive respiratory disease.

## 2. Case Presentation

### 2.1. Patient Information

A 26‐year‐old man was admitted on October 2, 2022 with intermittent dyspnea for 3 months that had worsened over the preceding 7 days. He also reported generalized weakness for 1 month and intermittent productive cough for approximately 6 months with whitish sputum. His body mass index was 14.45 kg/m^2^, indicating underweight status. However, he did not report recent weight loss. He denied chest pain, sore throat, anosmia, ageusia, night sweats, and significant weight loss.

### 2.2. Medical and Occupational History

The patient had been diagnosed with pulmonary TB on April 24, 2022 after GeneXpert detected *Mycobacterium tuberculosis* with rifampicin sensitivity. At the time of admission, he was in the fifth month of anti‐TB treatment and was receiving the continuation‐phase regimen. He also had a history of pneumothorax requiring chest tube insertion on May 2, 2022, which was removed on May 15, 2022.

He had worked regularly as a construction laborer since adolescence, for more than 10 years. Across successive construction projects, his tasks included cutting ceramic tiles, mixing cement, sanding building walls, and bricklaying, which he performed without respiratory protective equipment. He reported that work on an individual construction project could last approximately 1 year. However, the intervals between projects and the frequency and duration of individual dust‐generating tasks were unavailable. He also reported a smoking history of more than 10 years. Despite this history of unprotected occupational exposure, silica‐related lung disease had not previously been recognized, and his respiratory symptoms had initially been managed in the context of pulmonary TB.

### 2.3. Clinical Findings

On admission, he appeared weak but remained hemodynamically stable and fully conscious. His oxygen saturation was 90% on room air and improved to 99% with a simple mask at 6 L/min. His body mass index was 14.45 kg/m^2^. Physical examination showed dyspnea with use of accessory respiratory muscles and bilateral coarse crackles over the upper lung fields.

### 2.4. Diagnostic Assessment

Initial chest radiography showed a reticulogranular pattern in the parahilar, suprahilar, and right paracardiac regions, with bilateral suprahilar fibroinfiltrates (Figure 1). No chest radiographs obtained before the current presentation were available for comparison. The patient also had acute worsening of dyspnea and productive cough, bilateral upper‐lung crackles, leukocytosis (13,110/*μ*L), and neutrophilia (76.7%). Based on these findings, he was initially diagnosed with community‐acquired pneumonia, with a pneumonia severity index score of 56 (Class II).

**Figure 1:**
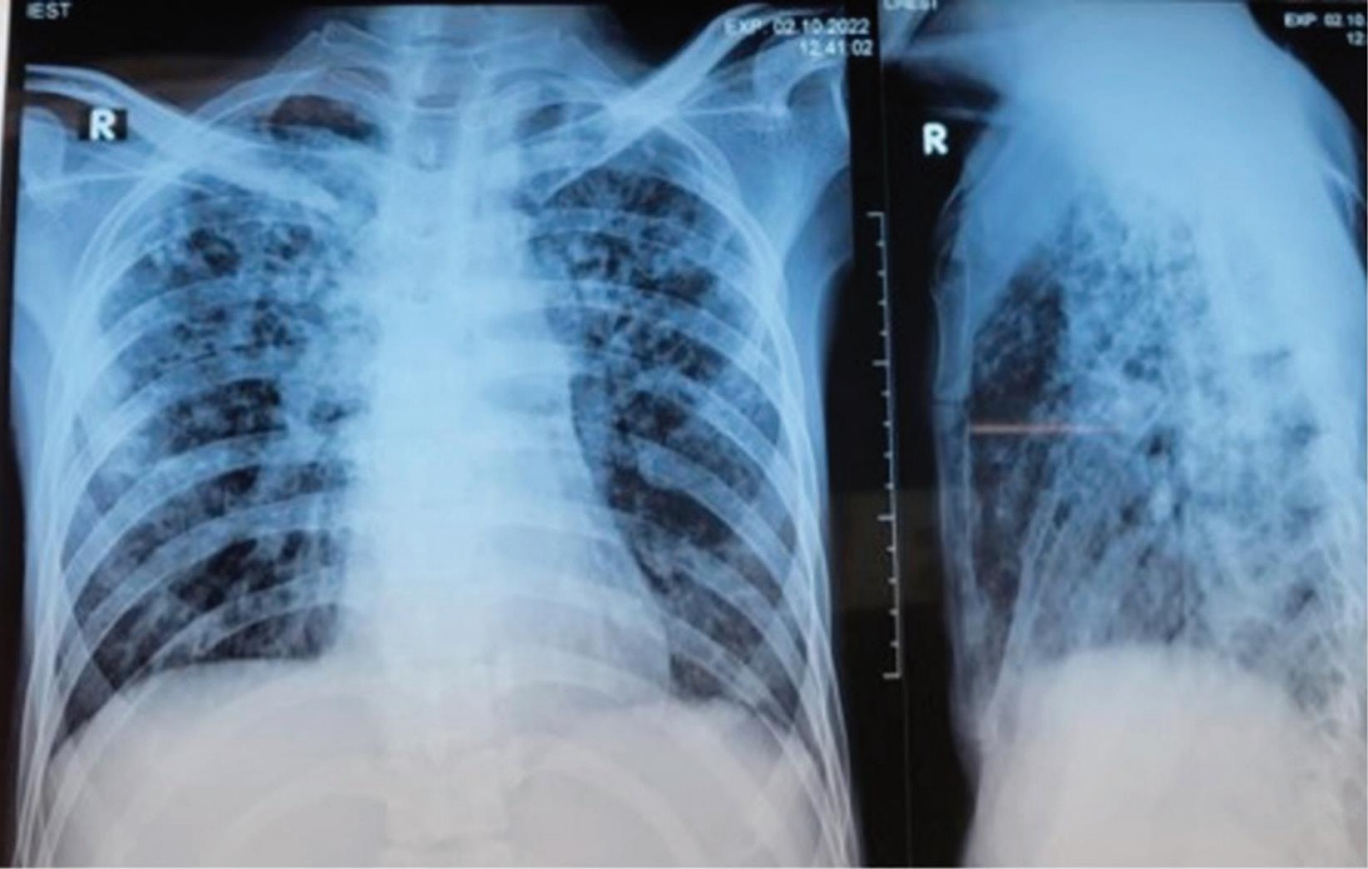
Initial chest radiograph showing reticulogranular opacities in the parahilar, suprahilar, and right paracardiac regions with bilateral suprahilar fibroinfiltrates.

During hospitalization, thoracic CT demonstrated bilateral fibroconsolidation with multitype bronchial dilatation, bilateral pleural thickening, and multiple calcified nodules in the apical segment of the right upper lobe (Figure 2). The calcified nodules were considered more likely to represent sequelae of previous pulmonary TB than a specific feature of silicosis.

**Figure 2:**
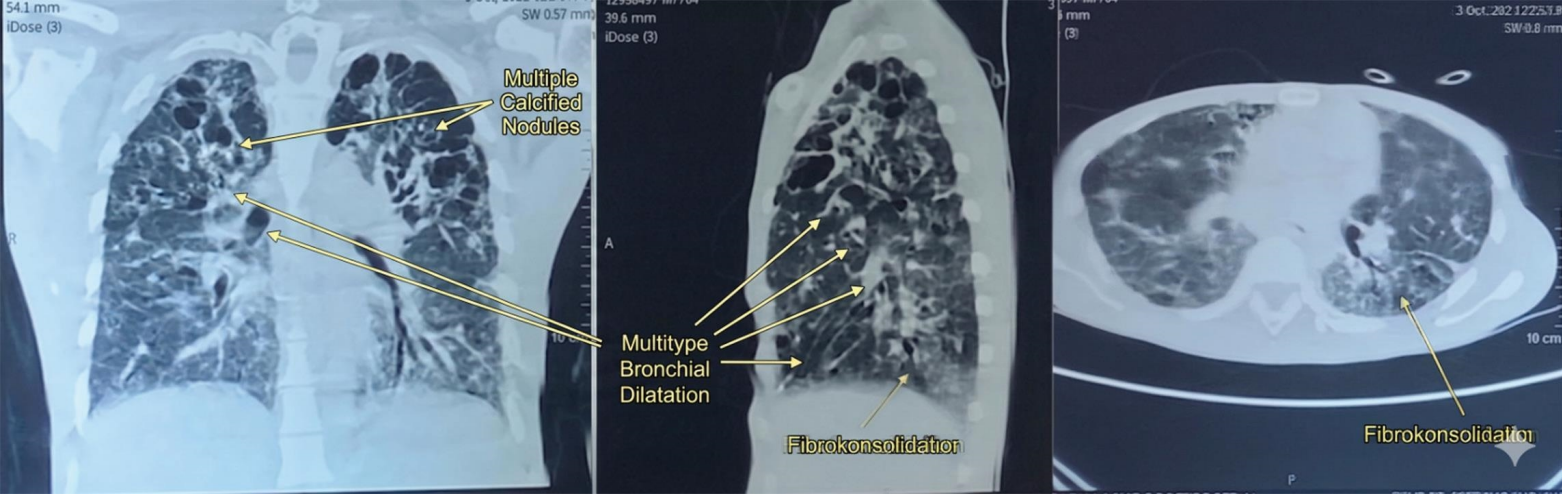
Thoracic CT showing bilateral fibroconsolidation with multitype bronchial dilatation and multiple calcified nodules in the apical segment of the right upper lobe, suggestive of silicotuberculosis.

Spirometry was performed on October 12, 2022 and showed a vital capacity and forced vital capacity of 0.50 L (approximately 12% of the predicted value of 4.06 L), suggesting severe restrictive impairment. However, the test was technically limited because the patient required supplemental oxygen and experienced repeated coughing during the maneuvers; therefore, the results should be interpreted with caution. Sputum smear for acid‐fast bacilli at Month 5 of treatment was negative. The persistence of respiratory symptoms and extensive radiologic abnormalities despite ongoing anti‐TB treatment prompted further evaluation for an underlying or alternative lung disease. HIV testing was nonreactive on October 2, 2022 and remained nonreactive on repeat testing on November 21, 2022.

### 2.5. Diagnostic Challenge and Procedure

Flexible bronchoscopy had initially been planned for further evaluation. However, the patient was considered high risk for general anesthesia because of oxygen desaturation without supplemental oxygen and persistent gas exchange impairment. Following multidisciplinary discussion, bronchoscopy was deferred and open lung biopsy with wedge resection of the posterior segment of the right upper lobe under intercostal block and local anesthesia was selected as an alternative diagnostic approach.

On October 18, 2022, the patient underwent thoracotomy through the right fifth intercostal space with open biopsy and wedge resection of the posterior segment of the right upper lobe. A lung tissue specimen measuring 3 × 5 cm was obtained, and a right chest drain was inserted.

### 2.6. Pathological and Tissue Analysis

Histopathological examination of the resected lung tissue showed pulmonary parenchyma with hemorrhagic areas and inflammatory infiltrates composed of lymphocytes, histiocytes, foamy macrophages, multinucleated giant cells, and neutrophils (Figure 3). Some alveoli showed fibrosis, and no evidence of malignancy was identified. Immunohistochemical examination demonstrated negative cytokeratin staining and positive CD68 expression in macrophages, consistent with chronic nonspecific inflammation. Although the histopathological findings were not specific for silicosis, the patient′s unprotected occupational exposure to silica‐containing dust, confirmed pulmonary TB, and overlapping radiological and pathological abnormalities were considered consistent with silicotuberculosis.

**Figure 3:**
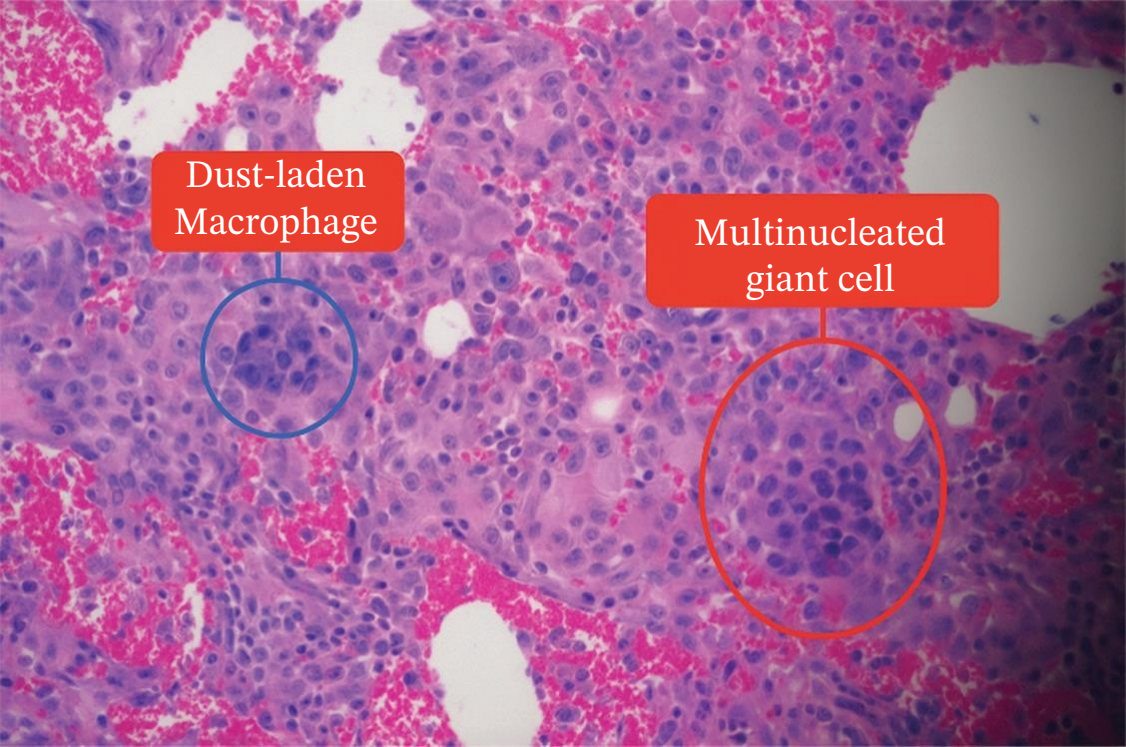
Histopathological examination of lung tissue biopsy showing multinucleated giant cell accompanied by chronic inflammatory cell infiltration and dust‐laden macrophages within the alveolar spaces, consistent with occupational dust exposure.

### 2.7. Treatment and Outcome

The patient received supportive oxygen therapy, continuation of anti‐TB treatment, empirical antibiotic therapy for community‐acquired pneumonia, chest physiotherapy, bronchodilator nebulization, mucolytic therapy, and correction of electrolyte imbalance. Sputum culture later grew *Aeromonas veronii*, whereas lung tissue culture grew *Pseudomonas* species, and antibiotic therapy was adjusted during hospitalization. His respiratory symptoms gradually improved during hospitalization. However, he remained oxygen‐dependent during mobilization. He was discharged on October 25, 2022 with long‐term oxygen therapy and continued outpatient anti‐TB treatment. He subsequently completed 6 months of treatment for rifampicin‐susceptible pulmonary TB. Anti‐TB treatment was stopped after 6 months following clinical improvement and negative sputum acid‐fast bacilli smears at months 2, 5, and 6. Treatment extension was not recommended by the infectious disease team. Despite the initial clinical improvement, he remained oxygen‐dependent during daily activities.

### 2.8. Follow‐Up and Prognosis

In November 2022, the patient was readmitted with worsening respiratory symptoms and was treated for bacterial pneumonia. He was subsequently discharged after treatment.

The patient did not attend further outpatient follow‐up because his clinical condition limited hospital visits. He was readmitted in May 2024 with worsening respiratory symptoms and received supportive treatment, including supplemental oxygen. Chest radiography on May 3, 2024 showed persistent bilateral fibroconsolidation, multiple calcified nodules in the right upper lung field, and multiple areas of bronchial dilatation, predominantly in the left lung (Figure 4). His condition continued to deteriorate, and he passed away during hospitalization in May 2024. No repeat chest imaging or pulmonary function testing was available.

**Figure 4:**
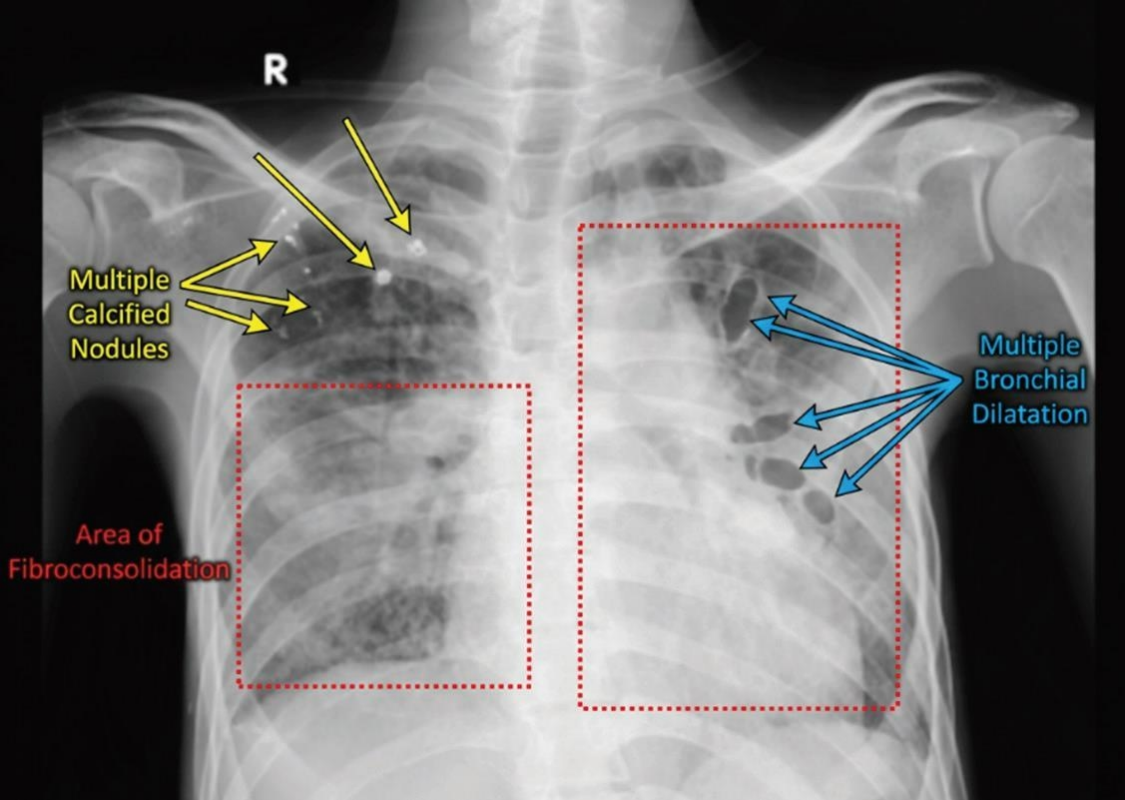
Follow‐up chest radiograph obtained on May 3, 2024 showing bilateral fibroconsolidation, multiple calcified nodules in the right upper lung field, and multiple areas of bronchial dilatation.

## 3. Discussion

The present case illustrates the diagnostic complexity of silicotuberculosis in a young construction worker with more than 10 years of unprotected occupational exposure to silica‐containing dust. Silicosis remains an important occupational disease, and hazardous exposure to respirable crystalline silica is increasingly recognized beyond traditional mining settings, including in the construction industry [9]. In this patient, unprotected occupational exposure to silica‐containing dust, microbiologically confirmed pulmonary TB, and overlapping radiological and pathological abnormalities were considered consistent with silicotuberculosis, although the respective contributions of silica‐related lung injury and TB could not be determined separately.

Although silicosis is classically associated with prolonged exposure in older workers, more recent reports have shown that silica‐related lung disease may also occur in younger workers, particularly when exposure is intense and poorly controlled [9]. As described earlier, the patient had worked in construction since adolescence and had more than 10 years of unprotected occupational exposure to silica‐containing dust. However, the intervals between projects, the frequency and duration of specific tasks, the use of wet or dry work methods, workplace ventilation, and other dust‐control measures were unavailable, precluding quantitative estimation of cumulative respirable silica exposure. The development of severe respiratory impairment at a young age was clinically compatible with an accelerated disease course [7]. However, because the radiological and histopathological findings were not specific for accelerated silicosis and could also reflect pulmonary TB, the overall presentation was better described as silicotuberculosis with an accelerated clinical course.

The conventional acute, accelerated, and chronic classification of silicosis is based largely on exposure intensity, latency, and clinical course [2, 7]. In this patient, histopathological examination did not show alveolar proteinosis or classic silicotic nodules. Moreover, the radiological and pathological abnormalities were not specific for accelerated or chronic silicosis and could also reflect pulmonary TB.

Silicosis is a well‐recognized risk factor for TB. Silica exposure has been reported to impair pulmonary immune defense and increase susceptibility to *M. tuberculosis*, providing a biologically plausible basis for this association [2]. Epidemiologic evidence further shows that individuals with silicosis have an approximately fourfold higher risk of TB than those without silicosis, whereas silica exposure in the absence of radiological silicosis is associated with an approximately twofold increased risk [6]. In the present case, unprotected occupational silica exposure is likely to have contributed substantially to the patient′s susceptibility to TB.

The patient completed 6 months of treatment for rifampicin‐susceptible pulmonary TB. Treatment was discontinued after clinical improvement and negative sputum acid‐fast bacilli smears at months 2, 5, and 6, and extension was not recommended by the infectious disease team. Although longer treatment has been proposed for silicotuberculosis because of the potentially increased risk of relapse, the available evidence remains inconsistent, and routine treatment prolongation is not included in recent expert consensus recommendations [10]. Microbiological response and treatment completion do not necessarily indicate resolution of TB‐associated inflammation or post‐TB structural lung injury. Possible interactions among occupational silica exposure, TB‐associated inflammation, and extensive structural lung damage may have contributed to the subsequent respiratory deterioration. However, no microbiological reassessment was available during the later deterioration, and it remains uncertain whether extended anti‐TB treatment would have altered the clinical course.

Establishing the diagnosis in this case was particularly challenging because the patient′s persistent pulmonary abnormalities could not be readily explained by treated pulmonary TB alone. Silicosis and pulmonary TB may coexist and produce overlapping clinical, radiological, and histopathological findings [2]. Diagnostic evaluation therefore depends on close correlation between occupational exposure history and imaging, whereas tissue sampling is usually reserved for cases in which noninvasive assessment is insufficient. Because bronchoscopy could not be performed safely in this patient, open lung biopsy was undertaken. Histopathological examination showed chronic nonspecific inflammation and focal alveolar fibrosis, findings that were not specific for silicosis and could not be clearly distinguished from changes associated with pulmonary TB. Nevertheless, the patient′s unprotected occupational exposure to silica‐containing dust, confirmed pulmonary TB, and overlapping radiological and pathological findings were more consistent with a silicotuberculosis phenotype.

The patient′s previous pneumothorax, severe restrictive impairment, and persistent oxygen requirement at discharge suggest substantial underlying structural lung damage. A similar severe pulmonary complication has also been reported in a young man patient with silicosis and prior TB who developed bilateral pneumothorax [11]. In the present case, wedge resection was performed primarily for diagnostic tissue sampling because bronchoscopy was not feasible. This differs from the report by Hapsari and Yudhawati, in which VATS wedge resection had a therapeutic role in bilateral primary spontaneous pneumothorax [12]. These cases illustrate that thoracic surgery may serve either diagnostic or therapeutic purposes in complex pulmonary disease.

The patient′s death during the final hospitalization is consistent with the poor prognosis reported in silicotuberculosis. Patients with silicotuberculosis have been reported to have higher risks of unfavorable treatment outcomes and death than patients with TB alone [1]. A recent survival analysis also found a twofold higher mortality risk and substantially shorter adjusted survival among patients with silicotuberculosis [13]. Although the immediate cause of death in this patient could not be confirmed, the combination of extensive structural lung abnormalities, pulmonary TB, chronic oxygen dependence, and recurrent pulmonary infection indicated a high risk of progressive respiratory deterioration.

This case highlights the preventable occupational component of silicotuberculosis and the need for broader social and regulatory action. In Indonesia, silicosis is formally recognized as an occupational disease, while workplace hazards are regulated under national occupational health and safety standards [14, 15]. Eligible workers with occupational diseases may receive protection and benefits through the Occupational Accident Security program [16]. However, the existence of these regulations and benefit schemes does not necessarily ensure effective enforcement, workplace inspection, or access to adequately resourced occupational surveillance. In this case, the available records did not document the patient′s employment arrangement, employer identity, workplace safety provisions, formal reporting as an occupational disease, referral for workplace investigation, or submission of a compensation claim. Therefore, it could not be determined whether these processes had been initiated or whether the patient and his family had accessed available benefits. In similar cases, formal reporting and referral should be considered to facilitate workplace investigation, preventive action, and access to eligible benefits.

This case underscores that occupational history taking should extend beyond job title and include a task‐based qualitative account of the materials handled, how dust was generated, the frequency and duration of specific tasks, the use of wet or dry work methods, workplace ventilation, dust‐control measures, and respiratory protection. When a severely ill patient cannot provide a prolonged history, the information may be supplemented by family members or obtained with support from trained occupational health or public health personnel. Pulmonary TB should not preclude further evaluation for an underlying occupational lung disease, particularly when the clinical course or imaging findings are not fully explained by infection alone. When noninvasive assessment is insufficient or cannot be performed safely, further imaging and tissue‐based evaluation may be required to clarify the diagnosis [2, 8]. Prevention should prioritize controlling respirable silica at its source through appropriate engineering and work‐practice measures. Respiratory protective equipment and TB and silicosis surveillance should complement, rather than replace, effective dust control. Screening programs should be adequately resourced and linked to workplace remediation, continued worker protection, occupational disease reporting, and access to eligible compensation [9, 10].

## 4. Conclusion

This case underscores the importance of recognizing silicotuberculosis as a combined disease process in workers with unprotected occupational exposure to silica‐containing dust and confirmed pulmonary TB. In this patient, the radiological and pathological abnormalities could not be attributed exclusively to either silicosis or TB, and the overall presentation was interpreted as silicotuberculosis with an accelerated clinical course. Early recognition of occupational exposure, prevention of further silica exposure, and appropriate management of TB and superimposed pulmonary infections are essential.

## Funding

No funding was received for this manuscript.

## Consent

Patient consent was obtained.

## Conflicts of Interest

The authors declare no conflicts of interest.

## Data Availability

Data sharing is not applicable to this article as no datasets were generated or analysed during the current study.
